# Current News and Opinion

**Published:** 1883-10

**Authors:** 


### Current News and Opinion.

### NO TIME FOR READING!

Accompanying remittances from our subscribers are many compliments and words of congratulation for the INDEPENDENT PRACTITIONER. One of the "old subscribers," however, acknowledges having received bills, and copies of the journal for a long time, but he "don't consider that he owes anything," because he "never gets time to read such things, or even to take them out of the *wrapers*." He also states that he has "writen" before to have it "stoped." (*sic.*)

Professional men may perhaps be excused when from lack of early opportunities both orthography and penmanship are at the lowest ebb. But for men utterly wanting in intellectual or literary attainments to persistently close their eyes to all avenues of improvement, little excuse can be offered.

The leading members of all professions amidst their many labors usually find time to read their periodicals and write for them, and many hold positions as college instructors besides. But there is a certain class of individuals who are unwilling to pay a trifling subscription fee, excusing themselves on the ground that they "have no time for reading." No time to keep up with the literature of their calling!

Such persons are the very ones who *ought* to take journals, and to profit by their teachings. C. E. F.

### ONE JOURNAL ONLY.

A distinguished member of our fraternity who "*talks* progress" loud enough, remarked at the recent meeting of the American Dental Association that "one dental journal if well boiled down" was all that was needed in the United States.

Goodness gracious, what a "progressive" idea! A single dental periodical for a nation!

Only literary ability enough in our whole country to stock a single monthly magazine? Only subscribers enough to maintain *one?*

We believe in getting as much light as it is possible to obtain from every source—all the good we can secure from every quarter. A single journal can hardly be expected to satisfy the demands of our whole body, even though well "boiled down." C. E. F.

---

### PREPONDERANCE IN SEXES.

According to Mr. Gosselin, Secretary to the British Embassy, Berlin, in an official report, he shows that London, in comparison with other cities, stands pre-eminent in the preponderance of females, the proportion being as 113.7 to 100. On the other hand, in Paris, in 1876, there were only 88.5 females to 100 males; in St. Petersburg (1881) 80.8, and in Rome (same year) 79.5.

If any one will, just at nightfall, take his stand in the lower part of Regent Street, or in the Haymarket, or upon the steps of the Criterion Restaurant, in Piccadilly, he will not dare dispute the statistician who affirms that, in London, the females are to the males in the ratio of ten to one, with wickedness in the same proportion.—EDITOR.

---

### DENTAL SURGERY.

Christopher Heath, the well-known English surgeon, says in a late address: "A most convincing proof of the scientific estimation in which dental surgery is now held, was given two years ago, when, at the International Medical Congress, which met in this city, the very active section of dental surgery contributed its full quota to the success of the meeting, and still more recently the Council of the

Royal College of Surgeons, of which I have the honor to be a member, has conferred upon one of the leaders of the dental profession the unsought honor of its fellowship, coupling the name of Mr. John Tomes with that of Professor Huxley, as representative men of science worthy of especial regard."

---

### SALIVARY CALCULUS.

Hyrtl, the anatomist, regards the tartar which collects on the teeth as a natural means intended for their preservation, the dentists to the contrary notwithstanding.—*Exchange.*

Hyrtl would probably regard dandruff as a natural means intended for the preservation of the hair; and dirt "a natural means intended for the preservation of the skin." What fools we mortals be.—Ed.

---

### AMERICAN ACADEMY OF MEDICINE.

The annual meeting of the Academy will be held at the New York Academy of Medicine, 12 W. 31st Street, New York, on Tuesday, October 9th, (three o'clock P.M.), and Wednesday, October 10th, 1883. Richard J. Dunglison,

Secretary.

---

### INODOROUS IODOFORM.

The peculiar odor of iodoform is found to be well masked by the addition of attar of rose, one minim to the drachm; or of essence of rose geranium, three or four minims to the drachm.—*Polyclinic.*

---

### DENTAL MEETING.

The Fifteenth Annual Union Meeting of the Seventh and Eighth District Dental Societies of the State of New York will be held in the City of Buffalo, on the 30th and 31st days of October next. The dentists of Western New York and of other localities are cordially invited to be present.

J. S. Walter, Sec. 7th Dist. Soc.

C. S. Butler, Sec. 8th Dist. Soc.

